# Antifungal Activity of Natural Compounds vs. *Candida* spp.: A Mixture of Cinnamaldehyde and Eugenol Shows Promising In Vitro Results

**DOI:** 10.3390/antibiotics11010073

**Published:** 2022-01-08

**Authors:** Ilaria Maria Saracino, Claudio Foschi, Matteo Pavoni, Renato Spigarelli, Maria Chiara Valerii, Enzo Spisni

**Affiliations:** 1 Microbiology Unit, Department of Specialized, Experimental and Diagnostic Medicine, Istituto di Ricovero e Cura a Carattere Scientifico St. Orsola Polyclinic, University of Bologna, 40138 Bologna, Italy; claudio.foschi2@unibo.it (C.F.); matteo.pavoni2@unibo.it (M.P.); 2 Department of Biological, Geological and Environmental Sciences, University of Bologna, 40126 Bologna, Italy; renato.spigarelli@studio.unibo.it (R.S.); chiaravalerii@hotmail.it (M.C.V.); enzo.spisni@unibo.it (E.S.)

**Keywords:** *Candida* spp., essential oils, cinnamaldehyde, eugenol, α-pinene, limonene, eucalyptol, antifungal properties

## Abstract

*Candida* spp. are commensal organisms of the skin, mucous membranes, gastrointestinal tract, blood, and vagina of animals and humans. In recent decades, the incidence of human fungal infections has increased, with *Candida* spp. (mainly *C. albicans*) infections being the most frequent, and the treatment of fungal infections is still a clinical challenge. Colonization of the human gastrointestinal tract by *Candida* spp. is significant because infections (e.g., candidemia and vulvovaginal candidiasis) frequently arise from commensal microorganisms. The aim of this study was to test in vitro the antifungal activity and the eventual synergistic effect of five pure components of essential oils: cinnamaldehyde, α-pinene, limonene, eucalyptol, and eugenol. These compounds were tested on 18 *Candida* strains (15 *C. albicans*, 2 *C. glabrata*, and 1 *C. lusitaniae*) derived from a culture collection of vaginal clinical strains. Methods: Fungistatic activity was evaluated using the disk diffusion method. For fungicidal activity, microdilution and time–kill curve protocols were set up. The checkerboard method was chosen to evaluate a possible synergistic effect of these compounds when mixed. Results: Cinnamaldehyde and eugenol gave the best results, inhibiting all the *Candida* strains and showing a highly additive effect (FICI 0.625). The cinnamaldehyde inhibition zone (IZ), MIC, and MFC means were 69 mm, 50.05 mg/L, and 109.26 mg/L respectively. Cinnamaldehyde led to the total loss of viable *Candida* cells within 4 h. Eugenol IZ, MIC, and MFC means were 35.2 mm, 455.42 mg/L, and 690.09 mg/L, respectively. Eugenol led to the total loss of viable fungal cells within 1 h. Treatment with α-pinene inhibited 88.9% of *Candida* strains, with an IZ mean of 21.2 mm, a MIC mean of 195.41 mg/L, and a MFC mean of 251.27 mg/L; this compound led to the total loss of viable fungal cells only after 24 h. Limonene inhibited only 33.3% of the tested strains and eucalyptol did not produce an inhibition halo, so these compounds were not tested further. Conclusions: These characteristics, together with the well-known safety of cinnamaldehyde and eugenol for human use, make these two natural compounds the perfect candidates for the treatment of candidiasis. This was a pilot study, the purpose of which was to evaluate the best composition of a mixture to be used against intestinal and vulvovaginal candidiasis; in vivo studies are needed to confirm these very encouraging results.

## 1. Introduction

*Candida* species are classified as yeasts belonging to the *Ascomycota.* They are commensal yeasts found on the skin, mucous membranes, gastrointestinal tract, blood, and vagina of animals and humans [1]. Since most fungi grow best at 20–30 °C, endothermic mammals with a core temperature higher than 37 °C are naturally resistant to systemic fungal diseases [2]. However, *Candida* spp. belong to the very few fungal genera able to grow at 37 °C and capable of causing disease in humans [3]. *Candida* spp. are polymorphic, growing as unicellular yeasts, multicellular pseudohyphae, or filamentous hyphae [4]; these features, together with the expression of adhesion proteins, hydrolytic enzymes [5] and their cell wall characteristics [6], act as virulence factors and have important roles in tissue penetration and invasion [7]. In recent decades, the incidence of human fungal infections has increased [8], with *Candida* spp. (mainly *C. albicans*) infections being the most frequent [9,10,11]. Colonization of the human gastrointestinal (GI) tract by *Candida* spp. is significant because *Candida* infections frequently arise from commensal organisms; in fact, in most cases of candidemia and vulvovaginitis caused by *C. albicans*, the strain identified in a patient’s blood/genitourinary tract sample and the strain identified in the same patient’s rectum sample are identical [12,13,14]. High levels of *Candida* spp. colonization were associated with several diseases of the GI tract, such as inflammatory bowel disease (IBD) and colorectal cancer (CRC), in which *Candida* species (especially *C. albicans*) overgrowth has been constantly observed [15,16,17,18,19,20]. *Candida* organisms also colonize gastric ulcers, causing slower healing [21,22]. All current studies agree on the fact that in the GI tract, inflammation promotes *Candida* colonization and, in turn, *Candida* colonization inhibits the healing of inflammatory lesions [23], thus inducing a vicious cycle that leads to the exacerbation of disease and can induce extra-gastrointestinal inflammation [24,25,26].

Vulvovaginal candidiasis (VVC) is an infection caused by *Candida* spp. that affects women of reproductive age and represents approximately 15–25% of vaginitis cases [27]. These microorganisms usually remain hosted in the vaginal mucous only as colonizers; however, under appropriate conditions, their growth rate increases, inducing the expression of virulence factors and affecting the mucous membrane [28]. Although most cases of vaginal candidiasis are mild, some women can develop severe infections [29,30,31]. The main etiological factor of vulvovaginal candidiasis is *C. albicans*, with a prevalence ranging from 85% to 95%. However, infections caused by other species, such as *C. glabrata*, *C. lusitaniae*, *C. tropicalis*, *C. krusei*, *C. parapsilosis*, and *C. kefyr*, are increasing because of single-dose treatment, low-dosage azole maintenance regimens, and the use of over-the-counter antimycotics that can induce drug resistance [30]. Recurrent vulvovaginal candidiasis (defined as four or more episodes of symptomatic infection within one year) [32,33] is usually due to a relapse from a persistent vaginal reservoir of organisms or endogenous reinfection (mainly from the gastrointestinal tract); the estimated probability of recurrent VVC by age of 50 ranges from 14 to 28% [34].

Essential oils (EOs) are volatile, complex mixtures of organic compounds obtained from leaves, flowers, fruits, seeds, roots, buds, stems, and wood through steam distillation [35,36]. Many EOs are commonly used as flavoring in food and drinks, and in cosmetic and pharmaceutical products [37]. EOs are known for their antibacterial, antifungal, antiviral, antioxidant, anticancer, and immune-modulatory activities; they are also used as analgesic, anti-inflammatory, spasmolytic, and locally anesthetic remedies [38,39,40,41,42].

Cinnamaldehyde, a 3-phenylprop-2-enal, is the main component (90%) of cinnamon essential oil. It is a yellow, oily liquid with a cinnamon odor and sweet taste. Cinnamaldehyde has a role as a hypoglycaemic, vasodilator, antifungal, and flavoring agent [43]. 

Pinene is a bicyclic, double-bond, terpenoid hydrocarbon, α- and β-pinene are the two isomers found in nature, e.g., in pine, rosemary (*Rosmarinus officinalis*), and *Satureja myrtifolia* essential oils. They are among the best-known representatives of monoterpenes, with various applications such as flavors and fragrances; they also show fungicidal, antiviral, and antimicrobial effects. In addition, α- and β-pinene are components of renal and hepatic drugs, and have been found to have inhibitory effects on breast cancer and leukemia cells [44].

Limonene (p-Mentha-1,8-diene) is a monoterpene found in the oils of Citrus plants like lemon, mandarin, orange, grapefruit, and bergamot. This 10-carbon cyclohexanoid monoterpene has two enantiomers, R and S. R is the main enantiomer in the essential oils of limonene; it can be used as a sweetener food additive and for fragrance in soaps and perfumes. It can be also used in the drug industry to facilitate the percutaneous transfer of drugs [45].

Eucalyptol (1,8-cineole) is a monocyclic terpene widely distributed in plants. The main food sources are eucalyptus oil (up to 80% eucalyptol), and herbs and spices such as mugwort, sweet basil, rosemary, sage, and cardamom. Eucalyptol is an ingredient of many brands of mouthwash and cough suppressant. It controls airway mucus hypersecretion and asthma via inflammatory cytokine inhibition. Eucalyptol is an effective treatment for nonpurulent rhinosinusitis, it reduces inflammation and pain when applied topically, and kills leukemia cells in vitro [46].

Eugenol (4-allyl-2-methoxyphenol) is an aromatic compound extracted from clove EOs that is used widely as a flavoring agent for foods and teas, as an herbal oil to treat toothache and to treat gastrointestinal and respiratory complaints (per *os*). Eugenol exhibits antibacterial, antineoplastic, anti-inflammatory, analgesic, and antioxidant properties, acting as radical scavenger, apoptosis inducer, voltage-gated sodium channel blocker, and NF-kB inhibitor [42,47].

Starting from these assumptions, the aim of this study was to assess in vitro the antifungal activities of five pure components present in different EOs: cinnamaldehyde, α-pinene, D-limonene, eucalyptol, and eugenol. All the active ingredients above listed are classified as GRAS (Generally Recognized As Safe) and thus can be used as ingredients in food supplements in appropriate dosages. They were tested on 18 *Candida* strains (15 *C. albicans*, 2 *C. glabrata*, and 1 *C. lusitaniae*) derived from a culture collection of vaginal clinical strains. These compounds were tested also on *L. acidophilus* to make sure that they would not inhibit healthy microbiota (at the concentration active against *Candida* spp.).

This was a pilot study, the purpose of which was to evaluate the best composition of a mixture to be used against intestinal and vulvovaginal candidiasis. 

## 2. Results

### 2.1. Disk Diffusion Method

**Figure 1:**
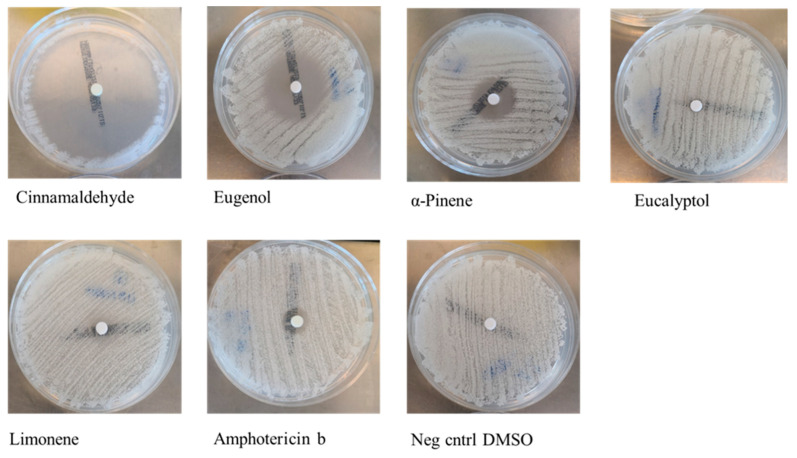
Representative pictures of agar diffusion assay.

The disk diffusion method was applied against 18 *Candida* strains (15 *C. albicans*, 2 *C. glabrata*, and 1 *C. lusitaniae*). The inhibition was considered successful when the inhibition zone (IZ) was equal to or wider than the amphotericin B inhibition zone (positive control). Eugenol and cinnamaldehyde inhibited all strains, α-pinene inhibited 88.9% of the tested strains, and limonene inhibited 33.3% of the strains, while eucalyptol did not produce an inhibition halo. These results are reported in Table 1 and Table 2 and Figure 1.

### 2.2. Microdilution Method

The antifungal activities of cinnamaldehyde, α-pinene, and eugenol were investigated using the microdilution method. Amphotericin B inhibition halos detected with disk diffusion were all in the susceptibility/intermediate susceptibility area, and these results were confirmed by microdilution. DMSO 1% (negative control) never inhibited cellular growth. Cinnamaldehyde showed the lowest MICs and MFCs. However, the MICs and MFCs of all three tested compounds were very low. The microdilution results are reported in Table 3 and Table 4. These compounds can be defined as fungicidal because the MFC/MIC ratio was <4.

### 2.3. Checkerboard Method

Cinnamaldehyde, α-pinene, and eugenol MICs for *C. albicans* strain 15 were, respectively, 40.95 mg/L, 268.13 mg/L, and 331.25 mg/L.

FICI 1: cinnamaldehyde in combination with eugenol. The first nonturbid well for the two combined oils was C8. In this well, the concentration of cinnamaldehyde was 20.48 mg/L, which was 1/2 of its original MIC, and the concentration of eugenol was 41.40 mg/L, which was 1/8 of its original MIC. The FIC index for these compounds was 0.625, corresponding to an additive effect. The suspensions derived from nonturbid wells were seeded, and the first spot without fungal growth was D9. The MFC of the mixture was 40.95 mg/L for cinnamaldehyde and 82.68 mg/L for eugenol.

FICI 2: cinnamaldehyde in combination with α-pinene. The first nonturbid well for the two combined oils was B9. In this well the concentration of cinnamaldehyde was 40.95 mg/L, which corresponded to its original MIC, and the concentration of α-pinene was 16.73 mg/L, which was 1/16 of its original MIC. The FIC index for these compounds when mixed was 1.0625, corresponding to an indifferent effect. 

FICI 3: eugenol in combination with α-pinene. The first nonturbid well for the two combined oils was B10. In this well, the concentration of eugenol was 662.50 mg/L, which was double its original MIC; the concentration of α-pinene was 16.73 mg/L, which was 1/16 of its original MIC. The FIC index for these compounds when mixed was 2.0625, corresponding to an indifferent effect.

### 2.4. Time–Kill Curve

**Figure 2:**
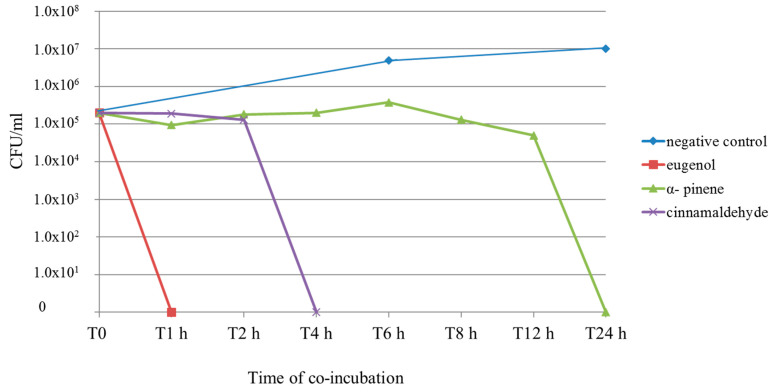
Time–kill curve for *C. albicans* strain number 15. Compounds were tested at their MFCs (2 × MIC in this case). MIC: minimal inhibitory concentration. MFC: minimal fungicidal concentration. CFU: colony-forming unit. Time is expressed in hours. Negative control: no compounds were added to the cell suspension.

**Figure 3:**
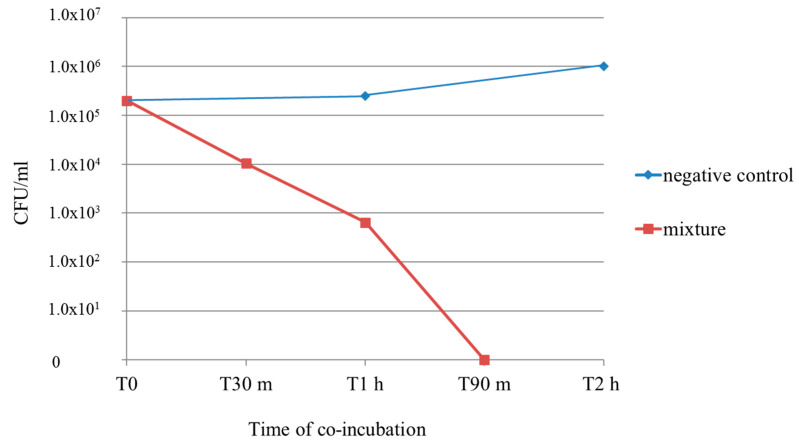
Time–kill curve for *C. albicans* strain number 15. The mixture of cinnamaldehyde/eugenol was tested at the MFC of the combination (2 × MIC of combination in this case). MIC: minimal inhibitory concentration. MFC: minimal fungicidal concentration. CFU: colony-forming unit. h: hours. m: minutes. Negative control: no compounds were added to the cell suspension.

A suspension of 2 × 10^5^ CFU/mL of *C. albicans* strain 15 was co-incubated with cinnamaldehyde, α-pinene, eugenol, and the mixture of cinnamaldehyde/eugenol at their MFCs. The total viable count (TVC) was determined at T0, T1 h, T2 h, T4 h, T6 h, T8 h, T12 h, and T24 h for the single compounds, and at T0, T30 min, T1 h, T90 min, T2 h, and T4 h for the mixture. Eugenol killed all fungal cells within the first hour of co-incubation. When *C. albicans* was co-incubated with cinnamaldehyde, the TVC was stable for 2 h, and at T4 h no more cells were viable. Effects of α-pinene were not detectable for the first 6 h, the TVC started to decrease at T8 h, and at T24 h all cells were killed. The combination of cinnamaldehyde/eugenol killed all fungal cells within 90 min. These results are reported in Figure 2 and Figure 3.

### 2.5. Antimicrobial Activity on Lactobacillus acidophilus

It is important that an antifungal substance does not affect the growth of healthy microbiota. When tested using the disk diffusion method, α-pinene and eugenol did not produce an inhibition halo for *Lactobacillus acidophilus*. Cinnamaldehyde created a 10 mm IZ. Evaluating the activity of cinnamaldehyde, the MIC against *L. acidophilus* was 327.60 mg/L, which was double the maximum MIC observed (only in 2 out of 18 strains) against *Candida* spp. and approximately 6.6 times greater than the mean MIC (50.05 mg/L). Evaluating the activity of α-pinene, the MIC against *L. acidophilus* was 1072.50 mg/L, which was double the maximum MIC observed (only in 2 out of 18 strains) against *Candida* spp. and about 5.5 times higher than the average MIC (195.41 mg/L). Evaluating the activity of eugenol, the MIC against *L. acidophilus* was 2650 mg/L, which was double the maximum MIC observed (only in 2 out of 18 strains) against *Candida* spp. and about 6 times higher than the average MIC (455.42 mg/L). From these findings, it can be deduced that the administration of cinnamaldehyde, α-pinene, and eugenol at their antifungal concentrations, leaves the normal microbiota unaltered. These results are reported in Table 5.

## 3. Discussion

*Candida* spp. are the most common members of human gut microbiota and are estimated to be present in 40–60% of the general population [48]. They may be present as transient or permanent colonizers in the oral cavity and in the further parts of the gastrointestinal tract, where they are capable of causing diseases. Fungal infections are commonly treated with antifungal drugs, mainly azoles and polyenes [6]. Azoles in particular are frequently associated with side effects and the prevalence of drug-resistant strains is increasing [9]; therefore, the treatment of fungal infections is still a clinical challenge [38]. The aim of this study was to evaluate the best composition of a mixture of natural compounds to be used against intestinal and vulvovaginal candidiasis. 

To assess the fungistatic activities of the five EO compounds, the disk diffusion method was used. The best results were obtained with eugenol and cinnamaldehyde, which inhibited all strains, and α-pinene, which inhibited 88.9% of the tested strains. Limonene showed a fungistatic activity in only 33.3% of cases, and eucalyptol did not produce an inhibition halo. From data reported in literature, both limonene and eucalyptol show antimycotic properties against *Candida* spp., mainly through interaction with ergosterol and inhibition of biofilm formation [49,50]. As regards the disk diffusion method, our results did not differ very much from those previously reported. Having established a criterion to consider the inhibition to be successful only when IZs were equal to or greater than those of amphotericin B, the fungistatic properties of limonene were not considered to be strong enough; moreover, eucalyptol inhibition was not detectable, and therefore the two compounds were not tested further in the subsequent experimental steps. Cinnamaldehyde and eugenol, on the other hand, produced the widest inhibition halos with mean diameters of 69 mm and 35.2 mm, respectively. 

For the second step of the study, the fungicidal activities of cinnamaldehyde, α-pinene, and eugenol were evaluated using the microdilution method. Cinnamaldehyde showed the lowest MICs (mean 50.05 mg/L) and MFCs (mean 109.26 mg/L). However, the MICs and MFCs of α-pinene and eugenol were also extremely low (MIC means 195.41 mg/L and 455.42 mg/L and MFC means 251.27 mg/L and 690.09 mg/L, respectively). 

Because it is known that the synergistic mechanisms occurring among the components of the essential oils are important determiners of their effects [51], we applied the checkerboard method, testing the three possible double combinations of eugenol, cinnamaldehyde, and α-pinene. The best results were obtained by combining cinnamaldehyde and eugenol, with a FICI of 0.625 corresponding to a strong additive effect. The combinations of cinnamaldehyde/α-pinene and eugenol/α-pinene showed indifferent effects. The best antifungal activity was obtained by mixing cinnamaldehyde at 1/2 of its MIC (20.48 mg/L) and eugenol at 1/8 of its MIC (41.40 mg/L). The MFCs of the two compounds when mixed were 40.95 mg/L for cinnamaldehyde and 82.68 mg/L for eugenol. 

To determine the kinetics of their fungicidal activity, the time–kill curve method was applied. *C. albicans* strain number 15 was chosen because the MICs and MFCs of the tested compounds for this strain were similar to the overall means, and the MFCs for this strain were always 2× the respective MICs (this ratio also remained stable for the combination). The *Candida albicans* n.15 total viable count was assessed at T0, T1 h, T2 h, T4 h, T6 h, T8 h, T12 h and T24 h. Eugenol killed all fungal cells within the first hour of co-incubation.

When *C. albicans* was co-incubated with cinnamaldehyde, the TVC was stable for 2 h, and at T4 h no more cells were viable. Effects of α-pinene were not detectable for the first 6 h, then the TVC started to decrease at T8 h and at T24 h all cells were killed. Eugenol exerted its fungicidal activity very quickly; this result is noteworthy and coherent with data reported in the literature [52]. 

The differences observed in the last two assays could be due to the fact that eugenol and cinnamaldehyde were able to permeate the cellular membrane, which represents the main target for the most common antifungal drugs and natural compounds, in a shorter time. In fact, cinnamaldehyde and particularly eugenol rapidly expressed their fungicidal effects in the time–kill curve assay, while α-pinene began to show its activity only after 8 h. Moreover, it led to an “indifferent” effect when combined with the other compounds; in particular, it caused a two-fold increase in eugenol MIC, for which our hypothesis is that α-pinene could have inhibited or temporarily delayed the permeability of the other compound or could have affected its access to cellular targets.

The combination of cinnamaldehyde and eugenol led to a rapid loss of viable cells; the count decreased from 2 × 10^5^ CFU/mL to 1 × 10^4^ CFU/mL within 30 min of co-incubation, at T1 h only 6.4 × 10^2^ CFU/mL were counted, and no more viable cells were detected at T 90 min. The mixture of cinnamaldehyde and eugenol killed all fungal cells in a slightly longer time than eugenol alone, but in a significantly shorter time than cinnamaldehyde alone. Nevertheless, it is important to underscore that when combined, these natural compounds expressed their antimycotic properties at much lower concentrations.

A further encouraging result obtained from in vitro assays was the absence of an antibacterial effect (at the concentrations active against *Candida* spp.) of these EO compounds against *Lactobacillus acidophilus*. *L. acidophilus* is the main component (90%) of the vaginal Doderlein complex, which forms a protective biofilm on the mucosa and inhibits the overgrowth of other microorganisms [53]; it has also been shown to reduce symptoms of ulcerative colitis in human patients [54] and reduce ulcer size in a rat model (ulcers induced by acetic acid + *C. albicans* inoculation) [22,55]. As mentioned above, both cinnamaldehyde and eugenol are defined as GRAS (Generally Recognized As Safe) with a DNEL (Derived No Effect Level) for the oral and dermal exposure in the general population of 0.625 and 3 mg/kg bw/day, respectively [56,57]. 

Cinnamaldehyde exerts its antimycotic activity mainly suppressing the ergosterol biosynthetic pathway [58] and simultaneously interacting with cell membrane by binding to ergosterol [59]. Cinnamaldehyde and its derivatives have also been reported to have a strong inhibitory effect on plasma membrane ATPase [58], and thus on ATPase-dependent efflux mechanisms. This leads to reduced proteinase and phospholipase activity and a suppressed secretion of hydrolytic enzymes. Plasma membrane ATPase also plays a regulatory role in *C. albicans* dimorphism [60]; therefore, its inhibition causes a significant decrease in germ tube formation, a pathological characteristic of *C. albicans* that facilitates invasion [61]. In addition, cinnamaldehyde is able to reduce *C. albicans* adhesion properties in vitro [62]. Eugenol exhibits antimicrobial activity towards several strains of pathogenic microorganisms [63]. It is known that eugenol also exerts its antifungal activity on *Candida* spp. mainly by inhibiting ergosterol biosynthesis and interfering with the integrity and functionality of the cell membrane. As a result, the functioning of membrane-bound enzymes such as those involved in cell wall synthesis is impaired, leading to damage to the cell wall [64]. The loss of structural integrity leads to increased permeability, which subsequently causes cell death [65]. As a matter of fact, it has been observed that eugenol causes a decrease in germination and elongation rates of *C. albicans* and suppresses cell growth and proliferation; treated *Candida* cells become flat and flaccid as a result of the change in hydroscopic turgor pressure [66]. 

Another noteworthy effect of both cinnamaldehyde and eugenol is their capability to reduce colonization and virulence factor expression of *Campylobacter jejuni*, a very harmful enteric pathogen [67]. 

Results obtained in this in vitro study suggest that a mixture of cinnamaldehyde and eugenol is suitable as a possible treatment for gastrointestinal and vulvovaginal candidiasis. In vivo studies are needed to confirm these encouraging results. For the treatment of *Candida* spp. overgrowth in the GI tract, it will be appropriate to test different formulations based on the site where action is needed, e.g., chewing gum, gastroresistant microcapsules, or nanocapsules. For vulvovaginal candidiasis, ovules containing the right proportions of these two EO compounds will be tested. 

## 4. Materials and Methods 

### 4.1. Natural Compounds

The essential oil individual components (≥98%) tested were cinnamaldehyde, α-pinene, limonene, eucalyptol, and eugenol. They were provided by Xeda International (S. Andiol, France). All other chemicals were of the highest purity and are commercially available.

### 4.2. Candida spp. Culture Conditions 

Eighteen *Candida* strains (15 *C. albicans*, 2 *C. glabrata*, and 1 *C. lusitaniae*) derived from a culture collection of vaginal clinical strains isolated on CHROMAgar Candida (BD Italy S.p.A, Milan, Italy) and confirmed via MALDI-TOF (Matrix-assisted laser desorption ionization-time of flight mass spectrometry, Bruker Daltonics S.R.L., Macerata, Italy) were cultured on Potato Dextrose Agar (PDA; Oxoid Thermofisher S.p.A, Milan, Italy) at 35 °C for 24 h. 

### 4.3. Antifungal Activity—Disk Diffusion Method

Colonies were suspended in sterile saline solution at a density corresponding to McFarland opacity standard #0.5 (*C. albicans* = 1.4 × 10^6^ CFU/mL, *C. glabrata* = 4.3 × 10^6^ CFU/mL, *C. lusitaniae* = 3 × 10^6^ CFU/mL) [68] to perform the susceptibility test to essential oils components. The agar disk diffusion method was carried out as follows: a total of eight Potato Dextrose Agar plates for every *Candida* strain were streaked in three directions with a swab dipped into each suspension to produce a lawn of growth. Blank filter paper disks (Oxoid Thermofisher S.p.A, Milan, Italy) with a diameter of 6.0 mm were added with 10 μL of each EO molecule and placed on the agar surface. Amphotericin B (Fungizone, Cheplapharm Arzneimitte srl; Greifswald, Germany, 10 μg) disks were used as positive controls. Clotrimazole (Sigma-Aldrich, St. Louis, MO, USA; from 10 μg up to 40 μg) disks were also used, but inhibition halos were not clearly readable due to the formation of a regrowth halo. Pure dimethyl sulfoxide (DMSO; Sigma-Aldrich, St. Louis, MO, USA; 10 μL) disks were used as negative controls. Plates were incubated at 35 °C for 24 h. All experiments were performed in duplicate. An EO susceptibility tests was considered positive if it resulted in an inhibition halo equal to or wider than that induced by amphotericin B (positive control). For amphotericin B, a ≥15 mm zone of complete inhibition was interpreted as “susceptibility”, from 10 to 14 mm as “intermediate susceptibility”, and <10 mm as “resistance” [69].

### 4.4. Antifungal Activity—Microdilution Method 

The antifungal activities of amphotericin B and of cinnamaldehyde, α-pinene, and eugenol (compounds with an inhibition halo equal to or wider than that of amphotericin B) were investigated using the microdilution method as reported by Bona et al. [51] with minor modifications. All 18 *Candida* strains were tested. A 24 h culture (in PDA) of each isolate was diluted in sterile saline solution to a concentration of 2 × 10^3^ CFU/mL (in order to obtain a final concentration of 10^3^ CFU/mL). Essential oils were dissolved in DMSO (80% oil, 20% DMSO), then diluted in Sabouraud broth to a concentration of 8% *v*/*v* (in order to obtain a final concentration of 4% *v*/*v*). Serial 1/2 dilutions of amphotericin B (range 32–0.032 mg/L, for a final range of 16–0.016 mg/L) and oils (range 8–0.004% *v*/*v*, for a final range of 4–0.002% *v*/*v*) were prepared in a 96-well microtiter plate. These %*v*/*v* ranges corresponded to 42 × 10^3^ to 20.48 mg/L for cinnamaldehyde, 42.4 × 10^3^ to 20.67 mg/L for eugenol, and 34.32 × 10^3^ to 16.73 mg/L for α-pinene. Each dilution of essential oils and amphotericin B (100 μL) was then inoculated with 100 μL of the cell suspension. Growth controls containing Sabouraud broth plus DMSO (prepared at 2% *v*/*v*, for a final concentration of 1% *v*/*v*) were also established. All microtiter plates were incubated at 35 °C for 48 h. Each experiment was repeated twice. MIC was determined as the concentration of the compounds in the first nonturbid well. MFC was determined as the lowest concentration of the compounds for which the subculture on agar showed the complete absence of fungal growth. The MIC of amphotericin B was also determined according to the EUCAST antifungal MIC method (cutoff 1 mg/L) [70]; all results were concordant.

### 4.5. Checkerboard Method

To investigate on the antifungal properties of these natural compounds when mixed, the checkerboard method on a 96-well microtiter was used.

First, 100 µL of Sabouraud broth was added to all wells except for H12. Next, 200 µL of the oil named A was added into the H12 well and 100 µL of the same oil (at 32 × MIC, for a final concentration of 8 × MIC) was added into wells A12 to G12. The compound was then two-fold diluted in a horizontal sense up to column 2. Additionally, 100 µL of the oil named B (at 16 × MIC for a final concentration of 4 × MIC) was added to wells H1 to H12, after which a two-fold dilution was carried out in a vertical sense up to row B. *C. albicans* strain number 15, from a 24 h culture on PDA, was suspended in Sabouraud broth at 2 × 10^3^ CFU/mL (for a final concentration of 10^3^ CFU/mL), and 100 µL of the suspension were dropped into each of the 96 wells. Microplates were incubated in at 35 °C for 48 h. MICs were determined as for the microdilution method. The A1 well was the negative control (without any substance).

FICI (fractional inhibitory concentration index) was determined as follows:

FICI 1: MIC (cinnamaldehyde in combination with eugenol)/MIC (cinnamaldehyde) + MIC (eugenol in combination with cinnamaldehyde)/MIC (eugenol)
FICI 2: MIC (cinnamaldehyde in combination with α-pinene)/MIC (cinnamaldehyde) + MIC (α-pinene in combination with cinnamaldehyde)/MIC (α-pinene)
FICI 3: MIC (eugenol in combination with α-pinene)/MIC (eugenol) + MIC (α-pinene in combination with eugenol)/MIC (α-pinene)

A synergistic effect is observed when FICI value ≤ 0.5; an additive effect when 0.5 < FICI value ≤ 1; an indifferent effect when <1 FICI value ≤ 4; and an antagonistic effect when FICI value > 4 [71,72]. 

For the combination of cinnamaldehyde/eugenol only, the nonturbid wells were seeded and MFCs of the compounds when mixed were calculated. All tests were performed in duplicate.

### 4.6. Time–Kill Curve (Fungicidal Activity) 

One *C. albicans* strain (number 15) was co-incubated with cinnamaldehyde, α-pinene, and eugenol at their MFCs. The *C. albicans* strain was suspended in Sabouraud broth at a density corresponding to McFarland opacity standard #1.4 (4 × 10^6^ CFU/mL), then diluted 1/10 (4 × 10^5^ CFU/mL, for a final concentration of 2 × 10^5^ CFU/mL). The suspension was divided into aliquots. A total of three aliquots were co-incubated with EO components (1:2 *v*/*v*) at 2 × MFC (for a final concentration = MFC); the fourth aliquot was diluted 1/2 with Sabouraud broth and was used as a negative control. The mixtures were thereafter incubated at 35 °C on a stirrer. At T0, T1 h, T2 h, T4 h, T6 h, T8 h, T12 h, and T24 h, 1/10 serial dilutions were performed to determine the viable count. From each dilution, 100 µL was subcultured onto PDA plates, which were incubated at 35 °C for 24 h. Plates were then inspected and the colonies were counted; their number was expressed as CFU/mL. The test was performed in duplicate. A time–kill curve was also determined for the cinnamaldehyde/eugenol mixture at the MFC of combination (calculated with the checkerboard method), and TVC was determined at T0, T30 min, T1 h, T90 min, T2 h, and T4 h.

### 4.7. Antimicrobial Effects on Lactobacillus acidophilus

The antibacterial activities of cinnamaldehyde, α-pinene, and eugenol against *L. acidophilus* were investigated using the disk diffusion and microdilution methods. One strain of *L. acidophilus*, namely La-14 (Danisco S.p.A., Milan, Italy), was tested. 

*Disk diffusion method*. A 24 h culture in DeManRogosaSharpe (MRS) agar was diluted in sterile saline solution at a density corresponding to McFarland opacity standard #0.5. A total of four MRS Agar plates were streaked in three directions with a swab dipped into the suspension to produce a lawn of growth. Filter paper disks (6.0 mm diameter) were placed on the agar surface and 10 μL of each compound was added. Pure dimethyl sulfoxide (DMSO; Sigma-Aldrich, St. Louis, MO, USA; 10 μL) disks were used as negative controls. Plates were incubated at 37 °C for 24 h in anaerobiosis (anaerogen gaspack, OXOID S.p.A., Milan, Italy). The experiment was performed in duplicate. 

*Microdilution method*. Essential oil components (80% oil, 20% DMSO) were diluted in MRS broth to a concentration of 8% *v*/*v* (in order to obtain a final concentration of 4% *v*/*v*). Serial 1/2 dilutions of the oils (range 8–0.004% *v*/*v*, for a final range of 4–0.002% *v*/*v*) were prepared in a 96-well microtiter plate. These % *v*/*v* ranges corresponded to 42 × 10^3^ to 20.48 mg/L for cinnamaldehyde, 42.4 × 10^3^ to 20.67 mg/L for eugenol, and 34.32 × 10^3^ to 16.73 mg/L for α-pinene. Each dilution (100 μL) was then inoculated with 100 μL of the cell suspension. All microtiter plates were incubated at 37 °C for 48 h in anaerobiosis (anaerogen gaspack, OXOID S.p.A., Milan, Italy). MIC was determined as the concentration of oils in the first nonturbid well. The experiment was performed in duplicate.

## 5. Conclusions

Cinnamaldehyde and eugenol showed high antimycotic activity against *Candida* spp. in vitro, in terms of both fungistatic and fungicidal properties. They had a strong additive effect when mixed and did not affect *Lactobacillus acidophilus* viability. These characteristics, together with their known safety, make these two natural compounds perfect candidates for the treatment of candidiasis. In vivo studies are needed to confirm these very encouraging in vitro results.

## Figures and Tables

**Table 1:** Summary of antifungal activities of selected natural compounds against *Candida* strains.

	IZ Diameter (mm)	% of Inhibited Strains
**AmphotericinB**		
mean	15.4	
range	10.0 to 20.5	
standard deviation	2.7	
**Cinnamaldehyde**		100% (18/18)
mean	69.0	
range	55.0 to 75.0	
standard deviation	6	
**α-Pinene**		88.9% (16/18)
mean	21.2	
range	14.0 to 3.01	
standard deviation	5.7	
**Limonene**		33.3 (6/18)
mean	13.9	
range	10.0 to 21.5	
standard deviation	3.2	
**Eucalyptol**	//	//
**Eugenol**		100% (18/18)
mean	35.2	
range	30.5 to 41.0	
standard deviation	3.5	
**Neg. control** **(DMSO)**	//	

The readings include the diameter of the paper disk (6 mm). IZ: inhibition zone. //: no inhibition halo detected.

**Table 2:** Antifungal effects of selected natural compounds against *Candida* strains evaluated by agar diffusion assay.

IZ Means (mm)						
	Amphotericin B	Cinnamaldehyde	α-Pinene	Limonene	Eucalyptol	Eugenol
*C. albicans* 1	13.0	65.0	27.0	14.5	//	35.0
*C. albicans* 2	15.5	70.0	17.5	10.5	//	35.0
*C. albicans* 3	16.0	67.0	23.5	12.0	//	33.0
*C. albicans* 4	19.5	66.0	25.0	12.0	//	35.0
*C. albicans* 5	16.0	71.0	23.0	17.5	//	35.0
*C. albicans* 6	16.0	67.0	24.0	12.0	//	36.0
*C. albicans* 7	16.0	75.0	30.0	21.5	//	44.0
*C. albicans* 8	20.5	67.0	31.0	14.0	//	41.0
*C. albicans* 9	12.0	70.5	14.5	14.0	//	34.0
*C. albicans* 10	15.0	72.5	19.5	14.5	//	34.5
*C. albicans* 11	14.5	75.0	15.0	12.0	//	34.0
*C. albicans* 12	16.5	75.0	15.5	13.5	//	34.0
*C. albicans* 13	16.0	71.0	20.0	12.0	//	35.0
*C. albicans* 14	19.0	75.0	14.0	13.5	//	34.0
*C. albicans* 15	15.5	70.0	15.5	14.0	//	34.0
*C. glabrata* 1	15.0	55.0	20.0	10.0	//	35.0
*C. glabrata* 2	11.0	55.0	16.5	12.0	//	30.5
*C. lusitaniae* 1	10.0	75.0	30.5	21.5	//	43.0

The reported results represent the means of four readings (the test was performed in duplicate, and for every halo the diameter was measured two times). The results include the diameter of the paper disk (6 mm). IZ: inhibition zone. //: no inhibition halo detected.

**Table 3:** Summary of the results from microdilution assay.

	MIC Mean	MIC SD	MIC Range	MFC Mean	MFC SD	MFC Range	MFC/MIC
Amphotericin B	0.052	0.04	≤0.016–0.125	0.11	0.08	≤0.016–0.25	
Cinnamaldehyde	50.05	36.78	≤20.48–163.80	109.26	102.50	≤21.00–656.25	2.18
α-Pinene	195.41	127.01	≤16.73–536.25	251.27	158.20	33.46–536.25	1.28
Eugenol	455.42	327.11	82.68–1325.00	690.09	424.10	165.36–1325.00	1.51

MIC and MFC distribution. MIC: minimal inhibitory concentration. MFC: minimal fungicidal concentration. SD: standard deviation. Unit of measurement: mg/L.

**Table 4:** Antifungal effects of selected natural compounds against *Candida* strains evaluated by microdilution assay.

	Amphotericin B		Cinnamaldehyde		α-Pinene		Eugenol	
	MIC	MFC	MIC	MFC	MIC	MFC	MIC	MFC
*C. albicans* 1	0.032	0.064	40.95	81.90	133.85	133.85	331.25	331.25
*C. albicans* 2	0.032	0.064	40.95	81.90	133.85	133.85	331.25	331.25
*C. albicans* 3	0.032	0.064	40.95	40.95	133.85	133.85	165.36	331.25
*C. albicans* 4	0.032	0.125	≤20.48	81.90	268.13	268.13	662.50	662.50
*C. albicans* 5	0.016	0.250	40.95	40.95	66.92	133.85	82.68	331.25
*C. albicans* 6	0.032	0.064	40.95	81.90	133.85	133.85	331.25	331.25
*C. albicans* 7	0.032	0.064	≤20.48	81.90	66.92	133.85	331.25	662.50
*C. albicans* 8	0.016	0.032	40.95	81.90	133.85	133.85	331.25	331.25
*C. albicans* 9	0.125	0.250	163.80	656.25	536.25	536.25	1325.00	1325.00
*C. albicans* 10	0.032	0.032	≤20.48	≤20.48	≤16.73	33.46	165.36	165.36
*C. albicans* 11	0.125	0.250	≤20.48	≤20.48	133.85	268.13	662.50	1325.00
*C. albicans* 12	≤0.016	0.016	≤20.48	≤20.48	≤16.73	66.92	165.36	662.50
*C. albicans* 13	0.064	0.125	81.90	163.80	268.13	268.13	662.50	1325.00
*C. albicans* 14	0.032	0.125	40.95	163.80	268.13	536.25	331.25	1325.00
*C. albicans* 15	0.032	0.064	40.95	81.90	268.13	536.25	331.25	662.50
*C. glabrata* 1	0.032	0.064	40.95	81.90	268.13	268.13	331.25	331.25
*C. glabrata* 2	0.125	0.125	163.80	163.80	536.25	536.25	1325.00	1325.00
*C. lusitaniae* 1	0.125	0.250	≤20.48	≤20.48	133.85	268.13	331.25	662.50

The test was performed in duplicate. When no well was turbid, the MIC was considered to be “minor or equal” to the minimal readable concentration. MIC: minimal inhibitory concentration. MFC: minimal fungicidal concentration. Unit of measurement: mg/L.

**Table 5:** Inhibition zones and MICs of cinnamaldehyde, α-pinene, and eugenol against *L. acidophilus*.

	IZ Mean (mm)	MIC Mean (mg/L)
Cinnamaldehyde	10	327.60
α-Pinene	//	1072.50
Eugenol	//	2650.00

IZ: inhibition zone. MIC: minimal inhibitory concentration. //: no inhibition halo detected.

## Data Availability

The data presented in this study are openly available in the Pub Med database.
